# Prediction of Maize Single Cross Hybrids Using the Total Effects of Associated Markers Approach Assessed by Cross-Validation and Regional Trials

**DOI:** 10.1155/2014/924348

**Published:** 2014-07-03

**Authors:** Wagner Mateus Costa Melo, Renzo Garcia Von Pinho, Marcio Balestre

**Affiliations:** ^1^Department of Agriculture, Federal University of Lavras, 3037 Lavras, MG, Brazil; ^2^Department of Exact Sciences, Federal University of Lavras, 3037 Lavras, MG, Brazil

## Abstract

The present study aimed to predict the performance of maize hybrids and assess whether the total effects of associated markers (TEAM) method can correctly predict hybrids using cross-validation and regional trials. The training was performed in 7 locations of Southern Brazil during the 2010/11 harvest. The regional assays were conducted in 6 different South Brazilian locations during the 2011/12 harvest. In the training trial, 51 lines from different backgrounds were used to create 58 single cross hybrids. Seventy-nine microsatellite markers were used to genotype these 51 lines. In the cross-validation method the predictive accuracy ranged from 0.10 to 0.96, depending on the sample size. Furthermore, the accuracy was 0.30 when the values of hybrids that were not used in the training population (119) were predicted for the regional assays. Regarding selective loss, the TEAM method correctly predicted 50% of the hybrids selected in the regional assays. There was also loss in only 33% of cases; that is, only 33% of the materials predicted to be good in training trial were considered to be bad in regional assays. Our results show that the predictive validation of different crop conditions is possible, and the cross-validation results strikingly represented the field performance.

## 1. Introduction

Maize breeding programs aim to create increasingly productive hybrids, which require conducting several crosses and generating several hybrids to be tested in trial networks. Although there are various breeding strategies, which range from population breeding to the creation and selection of haploids for the synthesis of lines, the choices of parentals and cross designs (factorial, diallels, triallels, or tetra-allels) are key issues to obtain high performance hybrids.

Although there are several methods available for planning crosses, the choice of method generally takes into consideration either the breeder's “experience,” desirable traits of the parental lines, and preestablished heterotic groups [1]. Although the breeder's “experience” can be highly useful, the choice of crosses based on additional information as pedigree data or genomic tools may be useful for directing crosses and even for increasing the predictive accuracy using optimum designs [2].

Another factor to be considered when choosing crosses is the number of lines to be used during hybrid synthesis. As the number of lines increases, the range of possible crosses can become so large that it economically precludes the establishment of all crosses [3]. Therefore, the breeder must unavoidably perform several crosses and disregard some potential crosses, which can result in several promising crosses not being performed and many inferior crosses being evaluated. Thus, the breeder must be able to predict all of the unevaluated crosses, even when evaluating only a portion of the crosses, to recover superior genotypes that would otherwise be discarded.

The first studies for predicting single cross hybrids using molecular markers were conducted using the genetic distance between lines. In a naïve view, the more divergent the parentals are, the greater the heterosis is expected to be because heterosis may be described by the genetic distance and the dominance effect when disregarding the epistasis [4].

Several studies have been conducted, starting in the 1990s, that aimed to predict the performance of hybrids based on genetic distance using this simplified theory of quantitative genetics [5–10]. However, the results found when using this approach were inconclusive and, in general, failed to reflect the credibility of its large-scale use in breeding programs.

New approaches were developed in parallel to the prediction using markers that were based on studies performed by [11] in mixed models context. [3] unified the analyses of mixed models and molecular markers in the prediction of single cross hybrids which used mixed models, complex pedigrees, and molecular markers [3]. The method consists of using a relationship matrix of the selection candidates in mixed model equations to recover unevaluated hybrids based on their genetic covariance with the tested hybrids. This method has been effective in predicting hybrids in various situations [12–15].

Recently, Schrag et al. [13, 16, 17] proposed the substitution of the genetic similarity matrix used in the Bernardo method with the genotype of the marker itself in the mixed model equations matrix. This new method was termed TEAM or total effects of associated markers. In this approach, the design matrix of general combining ability (GCA) and specific combining ability (SCA) is replaced by the additive and dominance matrix of molecular markers and ridge regression BLUP (RR-BLUP) [18] is used, wherein genetic values are assigned to each marker. The hybrid genotypic value can be recovered using the total effects of markers, as if through genomic selection. This method seeks to assess the genetic values of markers based on a test population and, subsequently, validate the prediction in the remaining genetic group, which may include both the untested hybrids and the test population itself, through cross-validation.

The TEAM method has proved to be more efficient than the method proposed by [3] in predicting hybrids [16]. More strikingly, [19] has extended this approach to the genomic wide selection (GWS) itself. However, normally the efficiency of TEAM or GWS approaches was not assessed by using real data set validation, that is, the data set where the predicted hybrids are assessed on different environments and years from those ones used in the training population. On the contrary, in traditional cross-validation method, a Jackknife procedure is used to assess the accuracy of the model where the same data set is divided in two groups of different sizes representing the training and validation population. As a result, the accuracy of model is tested by the use of the correlation between predicted and missing data. Therefore, the aim of the present study was to validate the TEAM method accuracy in the prediction of maize hybrids using cross-validation and regional data set in different environments and years.

## 2. Materials and Methods

### 2.1. Experimental Data

**Figure 1:**
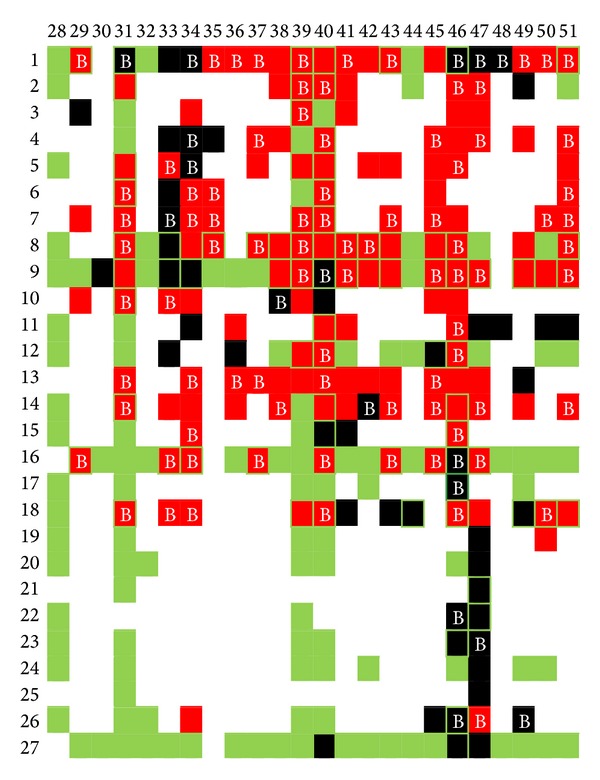
A diagram of the crosses conducted in the present trial (black) with the hybrids tested in regional assays (red). Hybrids selected for the test population are shown in green. Hybrids selected in the regional assay are represented by the letter B. The hybrids evaluated in the regional assay that were selected from the test population are outlined in green.

During the winter of 2010, 51 lines of different backgrounds were crossed to create the test population in the experimental field of Uberlândia South of Minas Gerais State, Brazil. Fifty-eight single cross hybrids were created starting from of these lines in an incomplete partial diallel system (Figure 1). These materials were evaluated in 7 locations distributed in the Southern region of Brazil (Vacaria, RS; Abelardo Luz, SC, Arapoti, PR, Candoi, PR, Canoinhas, PR, Castro, PR, and Ponta Grossa, PR) in 2010/2011 summer. Subsequently, in the 2011/12 summer, 119 new hybrids were generated from these 51 lines, for a final total of 175 genotypes to be evaluated during the second year of evaluation, which we regarded as the regional assays. These 175 hybrids were evaluated in regional assays conducted in 6 cities of Southern Brazil (Sananduva, RS; Vacaria, RS; Guarapuava, PR; Ipiranga, PR; Itapeva, SP; and Faxinal dos Guedes, SC). The conditions of the regional assays were identical to those described for the test population, although the year and locations were not necessarily the same.

The two experiments from both years were conducted using incomplete blocks designs with 2 replicates, and each plot had 4 to 5 m rows, with 70 cm spacing between rows. A complete description of this experimental design can be obtained at appendix notes in Melo et al. [20]. The grain yield was evaluated and adjusted for 13% moisture and converted into t*·*ha^−1^. The tillage and cover fertilizations were conducted according to the recommendations for each area, and cultural treatments were conducted to control the populations of Fall armyworm (*Spodoptera frugiperda*) and moth larva (*Helicoverpa zea*), as well as for weed control.

### 2.2. Molecular Data

Seventy-nine microsatellite markers were used to genotype the 51 lines (Table 1). These markers were distributed throughout the 10 linkage groups of maize.

The data matrix of markers was designed using the presence of allele *t* of marker *m* in line *i* as 1, and the absence of the allele was designated as 0. This coding scheme facilitated the design of the additive and dominance matrices of the hybrids.

Using the above coding scheme and considering that recombination is irrelevant in homozygous lines, the additive matrix of hybrids was designed as follows:

(1) $$\begin{array}{c} A_{lk}=\{\begin{array}{cc} 2, & \text{if}\;\;a_{1i}=a_{1j}=1,\\ 1, & \text{if}\;\;a_{1i}\neq a_{1j},\\ 0, & \text{if}\;\;a_{1i}=a_{1j}=0, \end{array}\;\forall a_{1i}\vee a_{1j}=\left\{1,0\right\}, \end{array}$$

where *a* is the genotype of the *t*th allele of marker *m* in the lines *i* and *j*, ∀ means for all situations where one or (∨) other lines present different alleles.

Similarly, the matrix of dominance effects was designed using the following relation (see [20, 21] for more details):

(2) $$\begin{array}{c} \Delta_{lk}=\{\begin{array}{cc} a_{1i}\times a_{1j}\vee a_{2i}\times a_{2l}, & \text{if}\;\;t_{i}=t_{j},\\ a_{1i}\times a_{2j}+a_{1j}\times a_{2i}, & \text{otherwise}, \end{array} \end{array}$$

for values of 1 and 2 of the *t*th alleles of marker *m*. A deviation of dominance for the homozygous allele occurs in the first case, and a deviation for heterozygous complement occurs in the second case.

### 2.3. Diallel Analysis and Total Effect of Associated Markers

The phenotypic data for the hybrids were analyzed using the partial diallel model and the markers model.

The following linear model was assumed in the first case:

(3) $$\begin{array}{c} y=X\beta +Z_{1}a_{1}+Z_{2}a_{2}+Z_{3}d+Z_{4}i_{1}+Z_{5}i_{2}+Z_{6}w+\xi , \end{array}$$

where *y* is the plot observation; *X* is the incidence matrix of fixed effects (blocks, replicates, and environments); *Z*_1_ to *Z*_6_ correspond to incidence matrices of random effects; the parameters *a*_1_, *a*_2_, *d*, *i*_1_, *i*_2_, and *w* correspond to the GCA effects of group 1 (GCA1), the GCA effects of group 2 (GCA2), the SCA, the interaction GCA1 × Environments, the interaction GCA2 × Environments, and the interaction SCA × Environments, respectively; and *ξ* is the model residue. Estimates of fixed effects, phenotypic variance components, and predictions of random effects were obtained by restricted maximum likelihood (REML), using the expectation-maximization (EM) algorithm.

The incidence matrices of parental effects and specific combination in the markers model were replaced by the matrices of additive and dominance effects of markers. Thus, the TEAM model was calculated as follows:

(4) $$\begin{array}{c} y=X\beta +Aa+\Delta d+\xi , \end{array}$$

where *A* corresponds to the matrix of incidence for additive effects (*a*) and Δ corresponds to the matrix of incidence for dominance deviations (*d*), both described above in molecular data topic. Unlike the RR-BLUP model, which is normally used in genomic selection, model 2 is characterized as a mixed model because the environmental effects are considered in the fixed effects matrix; furthermore, the interaction of the values assigned to each marker per environment was confounded with the residue due to high computational cost involved in estimating the interaction effects of each allele and the allelic interactions with the environment.

The genetic additive and dominance value of each hybrid *i* was recovered by the sum of additive effects of each allele within the specimen; that is, *α*_*i*_ = ∑_*j*=1_^*n*^ *λa*_*j*_ : *λ* = {0,1, 2} and *δ*_*i*_ = ∑_*j*=1_^*k*^ *ϕd*_*j*_ : *ϕ* = {0,1}, where *k* is the total number of allelic interactions *l* within each marker *m*, *n* is the number of alleles found in *m* markers, and *λ* and *ϕ* are indicator variables related to state of the marker *m* in the hybrid *i* for additive and dominance effects, respectively. It is equivalent to the point prediction *α*_*i*_ = *Α*_(*i*×*n*)_ *a* and *δ*_*i*_ = Δ_(*i*×*k*)_ *d* where *i* is the *i*th row of the matrices defined in (4).

Estimates of components of phenotypic variances and fixed effects were computed by predicting the additive and dominant effects contained in each marker through REML [20]. The total genetic variance recovered was regarded as common for each marker and was calculated as follows: *σ*_*g*_^2^ = *nσ*_*a*_^2^ + *kσ*_*d*_^2^ where *σ*_*a*_^2^ = (∑_*j*=1_^*n*^ *a*_*j*_^2^ + trace[*I*_*n*_ *W*_*n*_^−1^ *σ* ^2^])/*n* and *σ*_*d*_^2^ = (∑_*j*=1_^*k*^ *d*_*j*_^2^ + trace[*I*_*n*_ *W*_2_^−1^ *σ* ^2^])/*k*, where *W*_*n*_^−1^ and *W*_*k*_^−1^ are submatrices of the inverse matrix of the mixed model equations [22].

### 2.4. Cross-Validation and Validation Using Regional Trials

The cross-validation was performed using the set of hybrids that comprised the training population. For this purpose, different levels of imbalance were applied to the data set which corresponded to 58 single cross hybrids. The cross-validation was performed by resampling a group of individuals using the generalized Jacknife procedure [23]. The generalized Jacknife method is based on dividing the sample data set *C* into *g* groups of equal size *k*, so that *C* = *gk*. In each of the *g* groups, *k* individuals are removed to form the validation population.

The levels of imbalance ranged from 5 to 51%. The additive and additive + dominance models were used to predict the genotypic value of each hybrid. The correlation between the predicted and observed values was used as a parameter in the cross-validation. Raw molecular data were also used in the genetic distances as predictors of specific ability of hybrids. The genetic distance was calculated as 1 − *s*_*ij*_, where *s*_*ij*_ is Jaccard's genetic similarity.

In the second phase of the study, some hybrids combinations that were not tested in the training phase were predicted and compared with the results from the regional assays; that is, all possible combinations of the 51 lines were predicted based on results obtained from phase one, and those hybrids present in the regional assays were used to validate the method. The accuracy was assessed through quadratic regression fitting between the SCA predicted in the trial set and the values observed in the regional assays.

## 3. Results

The genetic variance of the general combing ability of group 1 was 0.055 (t*·*ha^−1^)^2^ (lines 1–27; Figure 1), while the genetic variance of group 2 was 0.077 (t*·*ha^−1^)^2^ (lines 28–51; Figure 1). The sum of the 2 GCA variances was 0.132 (t*·*ha^−1^)^2^, which was a larger value than that found for the variance of SCA, which was 0.063 (t*·*ha^−1^)^2^. The 3 combining ability variances recovered the total genetic variance calculated in the analysis of hybrids [0.22 (t*·*ha^−1^)^2^]. The heritability was 0.65, and the phenotypic variance was strongly affected by the interaction between the hybrids and the environments. The interaction between the hybrids and the environments was 3 times greater than the genetic variance and 5 times greater than the residual variance (*σ*_CGC*i*_^2^ = 0.45 t^2^ · ha^−2^ *σ*_CEC*i*_^2^ = 0.23 t^2^ · ha^−2^), indicating that the performance of the hybrids was highly affected by their interactions with the environments.

**Figure 2:**
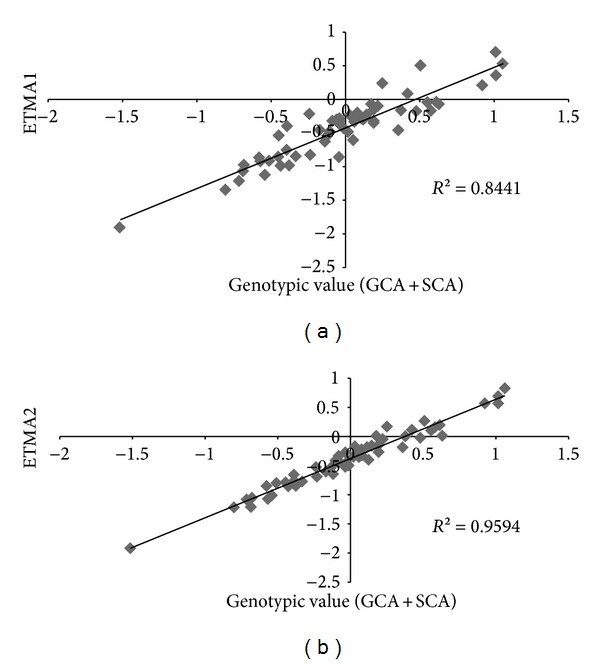
Total effects of the associated markers (TEAM) method when considering only the additive effect of markers (TEAM1) and the additive effects + the dominance effects (TEAM2).

The analysis using molecular markers in the lines showed 636 different alleles, with a mean of 8.05 alleles per locus. The crosses performed, shown in Figure 1, generated 1168 dominance effects; when these were added to the additive effects, there were a total of 1804 genetic effects that described nearly all of the genetic variance of the hybrids (*σ*_*g*_^2^ = *nσ*_*a*_^2^ + *kσ*_*d*_^2^ = 0.25 t*·*ha^−1^). This result can be confirmed in Figure 2, where the TEAM-2 model fit, which included the additive and dominance effects, was more effective in describing the performance of the hybrids than the TEAM-1 model, which only described the additive effects of markers. Figure 2 shows that the additive effects were more important than the dominance effects because the fit between the values predicted using the strictly additive model and the genotypic value was 0.84, whereas the fit that included the model of dominance effects became 0.95; that is, the adjustment of model was increased by 0.11 with the addition of the dominance effects. These results confirm the relationship between the magnitude of the GCA and the SCA variances calculated in the diallel analysis.

The process of cross-validation showed that the prediction ability of the TEAM-1 and TEAM-2 methods was reasonable to low, and the model including the dominance effects was slightly superior to the strictly additive model, as shown in Figure 2.  Table 2 shows the means, medians, and modes of the correlations between the predicted and observed values when considering the strictly additive model (TEAM-1) and the additive + dominance model (TEAM-2). These results show that as the number of hybrids removed increases (increased imbalance), the mode of the correlations between the observed and predicted values using the TEAM-1 model asymptotically tends towards the correlation value of 0.10 that was observed between genetic distance and grain production (Table 2, sample size 0*). This value was very low and nonsignificant according to the Mantel test. The TEAM-2 model, which we regarded as the additive + dominance model, was little affected by the number of imbalanced hybrids and maintained the most stable correlation, with the mode of the distribution of correlations remaining in the range of 0.34, even when more than 50% of the hybrids were discarded.

**Figure 3:**
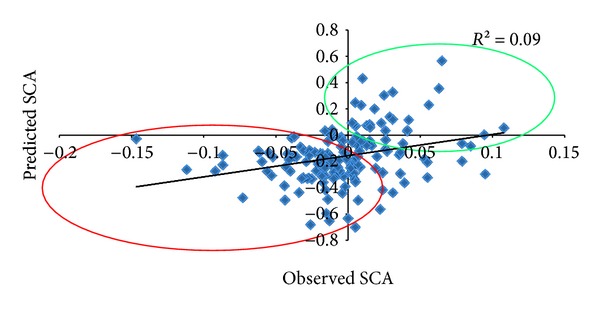
Credibility of the predicted values of the hybrids that were not tested by the TEAM2 method in the test population and the values recorded in the regional assays.

In the TEAM-2 model, the regression adjustment between the predicted hybrids and those observed in the regional assays was *R* ^2^ = 0.09 (Figure 3). Figure 3 shows that most of the high-performance hybrids observed in the regional assays were also those that were predicted to have high performance in training population (outlined in green), and the low-performance hybrids in the regional assays were also predicted to have low-performance in training (outlined in red). These results demonstrate that, in general, this method might be useful to discard the low-performance hybrids and selected the high-performance hybrids, even with a low fit.

**Figure 4:**
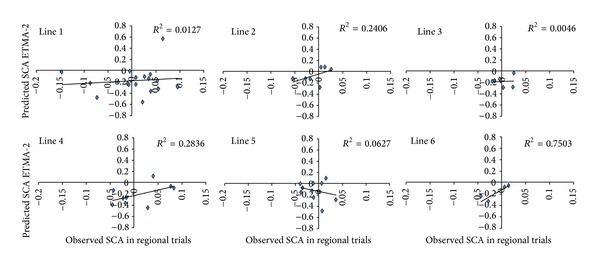
Total effects of associated markers (TEAM2) method fit per line with more than 4 crossing replicates (lines of 1–5).

**Figure 5:**
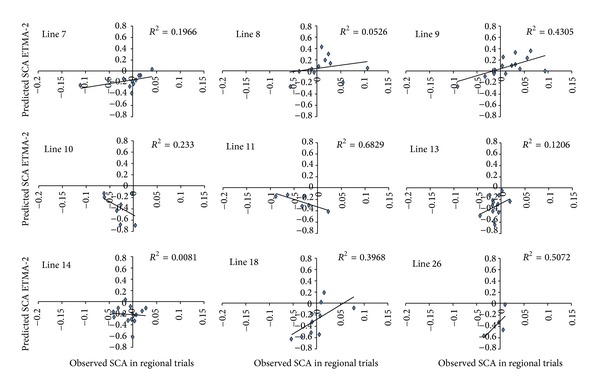
Total effects of associated markers (TEAM2) method fit per line with more than 4 crossing replicates (lines 7, 8, 9, 10, 11, 13, 14, 18, and 26).

Figures 4 and 5 show the predictive accuracy of hybrid performance per parental, considering the parental involved to be the genitors of at least 4 crosses. The results show that the prediction led to the selection of the best hybrids and the exclusion of the worst hybrids for most parental, with the exception of parentals 5, 10, 11, and 14. Figures 4 and 5 show that the predictive ability was high for crosses involving parentals 6 and 9 and was low for lines 10 and 11, suggesting that the method credibility may vary from line to line.

**Figure 6:**
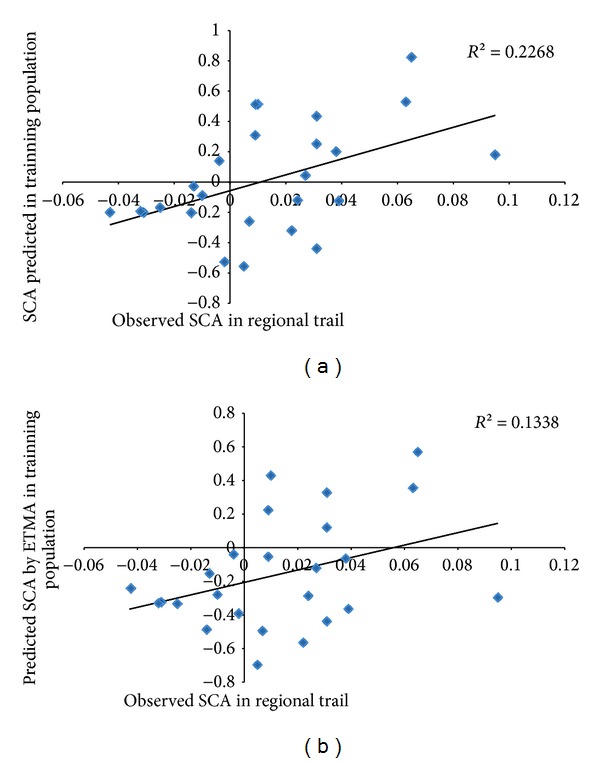
Selective credibility among the hybrids tested in the test population and the hybrids evaluated in the regional assay when considering the traditional method and the total effects of associated markers (TEAM2).

The response between the performance of the 58 hybrids tested in the training set (first-year tests) and their performance in the regional assays (second-year tests) is shown in Figure 6. On Figure 6(a) we have the measure of the credibility of the selection of hybrids at first year and their response at second year. On Figure 6(b) we have the predictive ability of TEAM-2 obtained for these 58 hybrids in training set, with the values observed in the regional assays. Considering that the hybrid heritability was 0.65 in the training population, the equivalent value for heritability would be expected to be repeated throughout the other environments because the SCA and GCA were free of interaction effects. However, Figure 6 shows that concordance among the performance of these 58 hybrids in the first and second year was just 0.23 and that the predictive ability dropped to nearly half (0.13) when using the performance predicted by the TEAM-2 method, a value rather close to the accuracy assessed between all 175 predicted hybrids and the values found in the regional assays (0.09; Figure 3). These results indicate that when the interaction between genotypes and environments is high, as was observed in the present study, the BLUP of hybrids cannot be extrapolated to any environment.

Figure 1 summarizes the whole dynamic of the present study, showing the hybrids tested in the first year (training population—black cells), the hybrids tested in the second year (regional assay—red cells), and the hybrids that were predicted to be good in the first year at 20% (green cells). Cells with the letter B refer to the selected hybrids selected in the second-year tests. Thus, those cells with the letter B that are marked in green refer to hybrids that were predicted to be good and that were confirmed as good in the second-year tests, that is, those hybrids where the selection was correct. Conversely, red cells marked in green without the letter B refer to hybrids that were predicted to be good but that were not selected in the second-year tests, that is, the selection error. Figure 1 clearly represents a 0 and 1 function loss; that is, 0 follows a correct selection (zero loss) and 1 follows an incorrect selection (total loss). The analysis of this parameter and a count of the cells in Figure 1 indicated a 50% correct prediction rate and a 33% error rate. That is, the method was able to correctly predict 50% of the hybrids selected in the regional assays; this is an extremely good value because the prediction was based on results from environments (locations of the first-year tests) that were extremely different from the environments used to validate the method (locations of the second-year tests). Furthermore, only 33% of the hybrids discarded in the regional assays were selected, which is an extremely good value considering the number of markers used and the number of hybrids tested. This result shows that this method is better for discarding the low-performance hybrids than for selecting the best hybrids.

## 4. Discussion

The genotypic values of SCA and GCA were broken down into 1804 additive and dominance effects, and the sum of those effects nearly recovered the genetic variance of the hybrids. The improvement in prediction using markers was not higher because the TEAM models did not include the interaction with environments, and that effect was confounded with the residual variance. The interaction between genotypes and environments was strongly involved in the phenotypic variance of hybrids and was the primary cause for the lack of genetic correlation between hybrids in the 6 locations evaluated in the second-year tests; therefore, the prediction of genotypic value might have been better if the interaction with the environment had been included.

The prediction results found in the present study are encouraging, considering the test population size (58 hybrids), the number of markers used (79 markers), and the completely different environments between the evaluation locations of the first- and second-year tests. For example, Schrag et al. [16, 17] found high values of *R* ^2^ (0.16–0.65) using an imbalance of 50% in a set of 400 tested hybrids and more than 1,000 molecular markers. Our results reached a fit of 0.15 for that level of imbalance using fewer than 10% of the number of markers and fewer than 15% of the number of hybrids in the test population than were used by Schrag et al. In contrast, in comparison to the study by [19], who obtained accuracy values of 0.75 to 0.87 for an imbalance of 10% when using cross-validation, our results reached 0.62 for that same level of imbalance and an accuracy of 0.95 with 5% imbalanced hybrids (3 hybrids excluded from the total of 58).

Another key result found in the present study was the ineffectiveness of using genetic distances to predict the performance of maize hybrids. There was only a 0.10 correlation between genetic distance and grain production, which is a nonsignificant value according to the Mantel test. Similar results were found by [9, 15, 24]. Thus, it may be inferred that it is better to use the data from markers as predictors in a linear model than to use genetic distances as predictors of hybrid performance because the amount of data contained in TEAM is far greater than the distances per se, even when using an imbalanced model.

Regarding the methods used to validate predictions, the studies published in the literature have usually used cross-validation as a method for assessing the accuracy of predictions and have not tested the predicted value of hybrids in conditions that differ completely from the test conditions; that is, under different environments and in competition with genotypes that are completely different from those used in cross-validation. Accordingly, the present study stands apart from all others by using the method efficacy to an extreme of prediction conditions and by considering that parameter for validating the method. The results found in the present study show that the accuracy of prediction for hybrid performance in locations and conditions of cultivation different from the test population is similar to that found under levels of imbalance above 30% (Table 1, Figure 3). Thus, cross-validation may be used under high levels of imbalance to assess the efficacy of the method in different conditions of cultivation.

Furthermore, the accuracy value was noticeably more stable than the credibility of selection assessed by using heritability because the estimated heritability was 0.65 and the realized was only 0.23. The value of 0.23 was calculated through the regression of the genotypic values of 58 hybrids tested in the training population and their genotypic values observed in the regional assays. The adjustment was 0.13, slightly above the adjustment for 175 hybrids that were not tested in the training population, which was 0.09 (Figure 3).

Another interesting result was the ability of the method to select hybrids correctly. A function of 0 and 1 type loss is reached when analyzing Figure 1, considering the value of 0 each time the correct hybrid was selected and the value of 1 each time the wrong hybrid was selected. Figure 1 clearly shows that 50% (30) of the best hybrids evaluated in the regional assay were selected using values predicted in the test population under a selection index of approximately 20%. Furthermore, only 33% (20) of the hybrids discarded in the regional assay were selected using values predicted in the test population. The TEAM method is clearly more efficient in avoiding type-1 errors (selecting the wrong hybrid) than type-0 errors (not selecting the correct hybrid), and the mode of distribution of cross-validation may be used in that case.

The present study demonstrated that the prediction of hybrids, especially maize, still requires further study. The increase in the number of markers and individuals in the test population may be crucial for validating the method. However, it became evident that the validation in regional assays, especially in locations and conditions of cultivation different from those adopted in the test population, may provide a wider perspective on the method efficacy to the breeder. Accordingly, the results from this study suggest that validation in different conditions of cultivation is possible, and the cross-validation results strikingly represent the field performance.

## Figures and Tables

**Table 1:** Distribution of the 79 microsatellite markers within the 10 linkage groups (LG) of maize.

Marker	LG	Bin	Marker	LG	Bin	Marker	LG	Bin	Marker	LG	Bin
bnlg1179	1	1.01	dupssr08	3	3.09	bnlg1200	7	7.01	umc1139	8	8.01
bnlg1014	1	1.01	bnlg1496	3	3.09	bnlg1808	7	7.02	bnlg1056	8	8.01
bnlg1007	1	1.02	umc1136	3	3.09	bnlg1305	7	7.03	bngl2082	8	8.03
bnlg1614	1	1.02	phi072	4	4.01	umc1342	7	7.04	bngl1067	8	8.03
bnlg1866	1	1.03	umc1101	4	4.09	bnlg2259	7	7.04	umc1858	8	8.04
umc1128	1	1.07	umc1109	4	4.10	umc1154	7	7.05	phi015	8	8.08
phi037	1	1.08	umc1197	4	4.11	umc1075	8	8.01	bnlg1131	8	8.09
bnlg1643	1	1.08	umc1058	4	4.11	umc1414	8	8.01	bnlg2122	9	9.01
umc1725	1	1.11	phi019	4	4.11	bnlg1194	8	8.01	umc1040	9	9.01
umc1797	1	1.12	umc1591	5	5.04	phi119	8	8.02	bnlg1724	9	9.01
umc1079	2	2.06	umc1482	5	5.04	umc1034	8	8.03	umc1078	9	9.05
bnlg1036	2	2.06	bnlg1237	5	5.05	phi115	8	8.03	umc1310	9	9.06
dupssr24	2	2.08	bnlg1118	5	5.07	mmc412	8	8.03	umc1319	10	10.01
bnlg1520	2	2.09	bnlg1371	6	6.01	umc2146	8	8.03	bnlg1079	10	10.03
umc1970	3	3.01	umc1006	6	6.02	phi121	8	8.03	umc2043	10	10.05
bnlg1601	3	3.05	umc1887	6	6.03	umc2147	8	8.03	bnlg1074	10	10.05
bnlg1160	3	3.06	umc1918	6	6.04	umc1157	8	8.03	umc1061	10	10.06
umc1148	3	3.07	bnlg1740	6	6.07	umc1202	8	8.05	bnlg1360	10	10.07
umc1167	3	3.08	phi089	6	6.08	bngl240	8	8.06	umc1084	10	10.07
bnlg1108	3	3.08	umc1066	7	7.01	umc1933	8	8.08			

**Table 2:** Means, medians, and modes of the correlations between the predicted values of untested hybrids and the outcomes of the test population, using different sample sizes of disequilibrium in both the additive and additive + dominance models.

Size and percentage of missing hybrids	Additive			Additive + dominance		
	Mean	Median	Mode	Mean	Median	Mode
3 (5%)	0.28	0.51	0.95	0.39	0.68	0.94
6 (10%)	0.36	0.44	0.53	0.38	0.47	0.62
9 (15%)	0.21	0.30	0.44	0.36	0.35	0.32
12 (20%)	0.28	0.26	0.26	0.35	0.36	0.37
15 (25%)	0.26	0.29	0.39	0.32	0.32	0.34
17 (30%)	0.25	0.23	0.21	0.30	0.33	0.40
20 (35%)	0.22	0.24	0.27	0.29	0.30	0.39
25 (43%)	0.22	0.23	0.23	0.27	0.27	0.34
30 (51%)	0.15	0.14	0.09	0.27	0.28	0.34
∗	0.10^ns^			0.07		

*Correlation assessed by genetic distance; ^ns^nonsignificant according to the Mantel test.
